# Diagnostic Accuracy of Multidetector Computed Tomography in Detection of Esophageal Varices

**DOI:** 10.7759/cureus.3933

**Published:** 2019-01-21

**Authors:** Maria Hassan, Yousuf Husen, Summar-un-nisa Abbasi, Zainab Hussain

**Affiliations:** 1 Radiology, Dr. Ziauddin Hospital, Karachi, PAK; 2 Radiology, The Agha Khan University Hospital, Karachi, PAK; 3 Radiology, The Aga Khan University Hospital, Karachi, PAK

**Keywords:** multidetector computed tomography, esophageal varices, mdct

## Abstract

Objective

To determine the diagnostic accuracy of multidetector computed tomography (MDCT) in the detection of esophageal varices by taking endoscopy as the reference standard.

Materials and methods

This was a cross-sectional prospective study conducted at the Department of Radiology, Aga Khan University Hospital, (AKUH) Karachi, for the duration of 12 months from August 1, 2014 to July 31, 2015. One hundred ninety-six patients with a suspicion of chronic liver disease/cirrhosis undergoing 64 slice MDCT were enrolled in our study and underwent computed tomography (CT) scanning in the Department of Radiology at AKUH. Biphasic CT was performed with images obtained during the hepatic arterial phase (30-second delay) and the portal venous phase (65-second delay) after the intravenous (IV) injection of 120 mL of nonionic contrast material at a rate of 3.5 mL/s. The presence of esophageal varices was evaluated on MDCT with endoscopy as gold standard. The sensitivity, specificity, negative predictive value and positive predictive value, and accuracy of MDCT were assessed against the gold standard.

Results

Our results yielded an MDCT sensitivity of 98.96%, specificity of 100%, positive predictive value (PPV) of 100%, negative predictive value (NPV) of 66.67%, and diagnostic accuracy of 98.97% for esophageal varices in chronic liver disease (CLD) patients.

Conclusion

The rate of detection of esophageal varices in patients with chronic liver disease on MDCT in our country is comparable to the international data and we advocate that MDCT should be used as a screening tool in patients with chronic liver disease to exclude esophageal varices.

## Introduction

Chronic liver disease and cirrhosis is a worldwide disease accountable for significant morbidity and mortality [1]. In Pakistan, its incidence is on the rise mostly due to chronic hepatitis B and C infections, the prevalence of which is reported to be up to 4.3% and 4.7%, respectively [2-4].

During their entire lifetime, at least 50% of cirrhotic patients develop esophageal varices [5]. Life-threatening upper gastrointestinal (UGI) bleeding due to the esophageal varices and as a complication of portal hypertension develops in about 30%–40% of cirrhotic patients. These patients have a 10%–30% risk of variceal haemorrhage per year leading to increased morbidity and mortality with a mortality rate of 20%–35% at their first bleeding episode [6-7]. Considering the morbidity and mortality associated with variceal haemorrhage, it has been recommended that these patients should undergo screening endoscopy for early diagnosis and treatment for variceal haemorrhage. Prophylactic endoscopic and medical interventions are offered to these patients so that the associated morbidity and mortality can be reduced [7].

Over time, it has been observed that screening endoscopy is invasive, expensive, needs sedation, and is sometimes poorly tolerated by the patient [8]. However, with recent advancement in multidetector computed tomography (MDCT), the role of CT has expanded for the surveillance of chronic liver disease patients. CT is approximately 90% sensitive and 50% specific in the detection of esophageal varices, hence CT can be considered as a potential noninvasive and less expensive modality for esophageal varices identification and risk stratification [8].

The ability to directly visualize esophageal varices on cross-sectional imaging has long been known, although systematic evaluations of diagnostic accuracy are mostly recent and few in number. Liver multidetector computed tomography (MDCT), which is often performed for hepatocellular carcinoma (HCC) screening and surveillance of cirrhotic patients, provides good coverage of the distal esophagus and hence can also be useful for the diagnosis of esophageal varices [9].

This may enable us to detect those patients who can benefit from early prophylactic intervention and subsequently reduce associated morbidity and mortality. In addition, we may also save patients from invasive, less compliant, and more expensive endoscopy. To our knowledge, multiple international studies have been done on the accuracy and grading of esophageal varices with liver CT; however, not much local literature is available despite the increased incidence of chronic liver disease and cirrhosis in our country.

The objective of this study was to determine the diagnostic accuracy of multidetector CT in the identification of esophageal varices by taking endoscopy as the reference standard.

## Materials and methods

A cross-sectional prospective study was conducted at the Department of Radiology, Aga Khan University Hospital (AKUH), Karachi, from August 1, 2014 to July 31, 2015 for a duration of 12 months.

The reported sensitivity and specificity of MDCT to detect esophageal varices is 90%^ ^[6] and 50% [6], respectively. The prevalence of esophageal varices in chronic liver disease is 50% [3]. A total of 196 patients were included in the study. All the patients with known chronic liver disease / cirrhosis referred to the radiology department at AKUH for MDCT, followed by endoscopy within 20 days were included in the study. The exclusion criteria included already diagnosed cases of esophageal varices, patients with incomplete medical records, those who did not undergo endoscopy after MDCT, and those who underwent endoscopy after 20 days. Informed consent was taken. All patients underwent MDCT for the screening of hepatocellular carcinoma. The endoscopy findings of these patients were acquired from the hospital’s medical record system.

All the CT imaging was performed on a system with 64 slice multidetector computed tomography (Aquilion 64, Toshiba Medical Systems Corporation, Otawara, Japan). KVp and mAs were set using automatic exposure control. (Automatic exposure control is a default setting in the equipment to control the exposure and is calibrated every three months by an experienced physicist with a minimum of five years of experience.) CT images were obtained during the hepatic arterial dominant phase using a 30-second delay and the portal venous dominant phase using a 65-second delay after the initiation of intravenous (IV) injection of 120 mL of nonionic contrast material at a rate of 3.5 mL/s. First, volume data with 0.5 mm slice thickness was acquired followed by reconstruction in the coronal and sagittal planes with slice thickness of 5 mm and 3 mm, respectively.

The imaging interpretation was performed by an experienced consultant radiologist with a minimum of five years of experience in body imaging, who was blinded with the endoscopy findings. Endoscopy was performed by an experienced consultant gastroenterologist with a minimum of five years of experience who was blinded with the CT findings. Endoscopy served as the reference standard.

Statistical data analysis was done using the Statistical Package for the Social Sciences (SPSS version 20) (IBM Corporation, USA). Sensitivity, specificity, positive predictive value (PPV), negative predictive value (NPV), and accuracy were calculated for the selected criteria. Positive cases included true positive and false negative; and negative cases included the true negatives and the false positive cases. Mean and standard deviation were calculated for the age of the patient. Frequency and percentage were also calculated for the gender of the patients. The effect modifiers hepatitis B and hepatitis C were controlled in the data analysis.

## Results

We included 196 patients: 106 were men (54.08%) and 90 were women (45.91%). A wide range of age was seen and it varied between 11 to 85 years with a mean age of 55.84 years.

**Figure 1:**
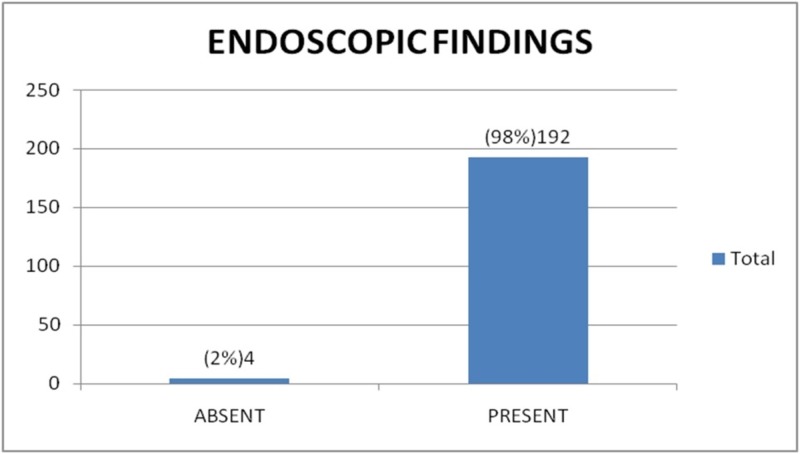
Endoscopic findings

**Figure 2:**
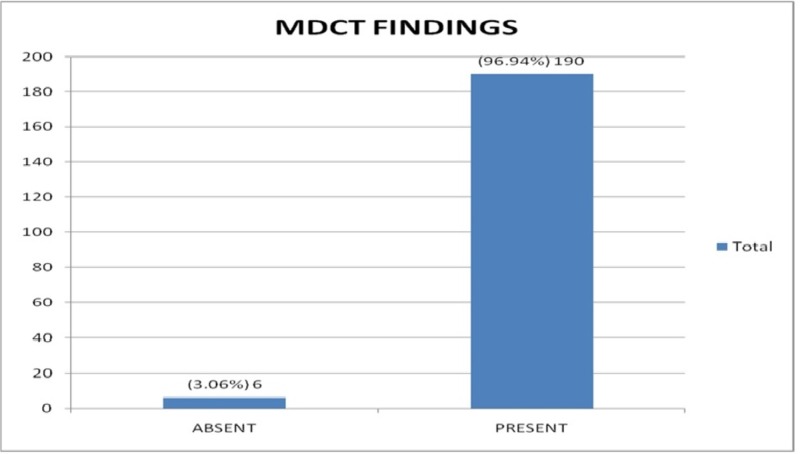
MDCT findings MDCT - multidetector computed tomography

**Figure 3:**
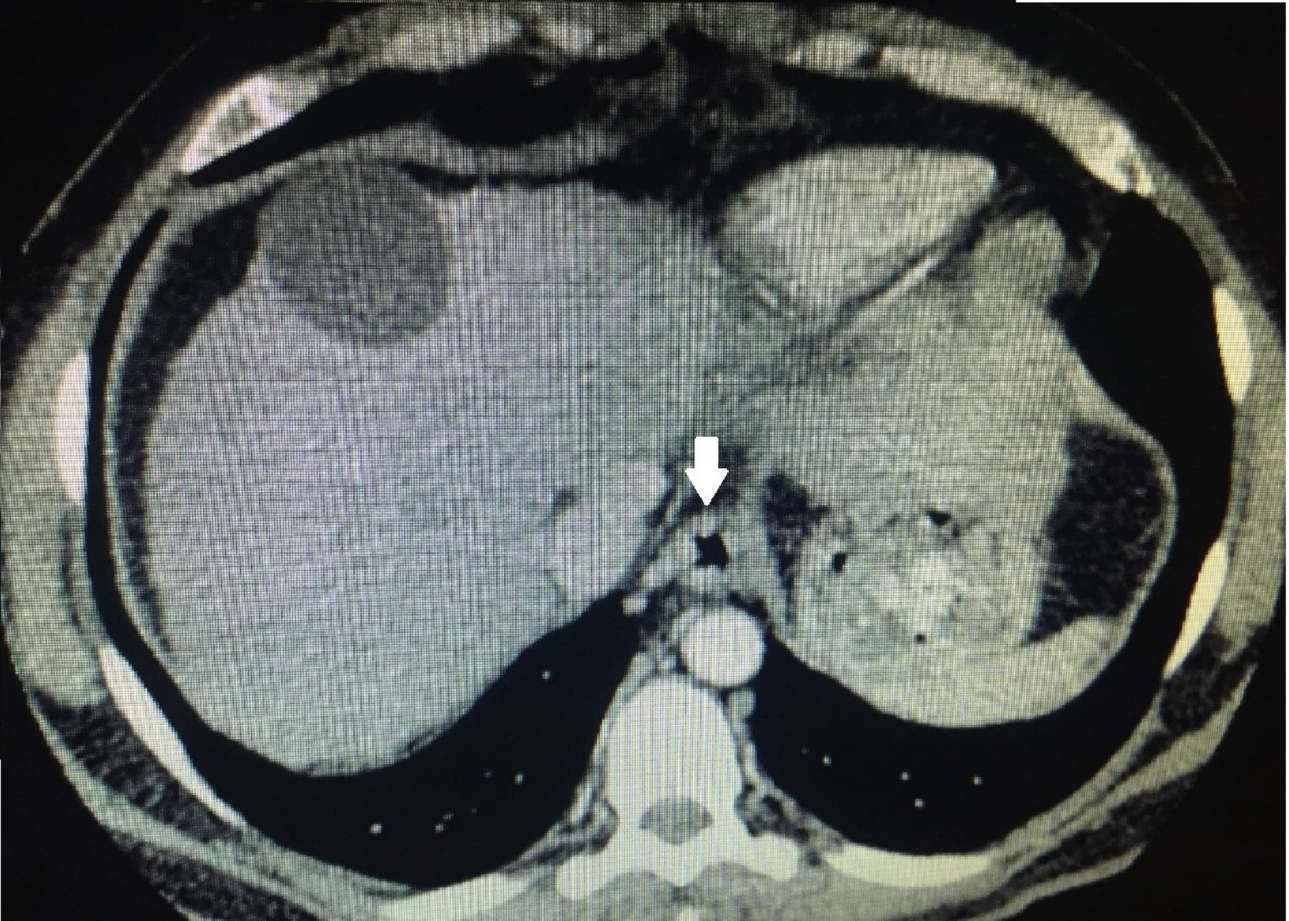
MDCT axial post contrast image of a patient with chronic liver disease showing nodular mucosal enhancement of esophagus representing esophageal varices

Out of the 196 cases, 192 cases (97.9%) were identified by endoscopy as having esophageal varices and four (2.04 %) were reported as not having esophageal varices (Figure 1). Later on MDCT, 190 (96.9%) of the total 196 cases were found to have esophageal varices while six (3.06%) were reported to not have esophageal varices (Figures 2-3). There were 190 true positive, zero false positive, four true negative, and two false negative cases on MDCT with endoscopy taken as a gold standard for the diagnosis of esophageal varices (Table 1).

**Table 1:** 2 X 2 table showing true positive, false positive, true negative and false negative

	ENDOSCOPY FINDINGS	
MDCT FINDINGS		
	Varices (+VE)	Varices (-VE)
Varices (+VE)	190 (a)	0 (b)
Varices (-VE)	2 (c)	4 (d)

Among the 196 cases, 79 cases were hepatitis C positive, 13 were hepatitis B positive and the rest had other causes of cirrhosis. Based on these findings, MDCT has a sensitivity of 98.96%, specificity of 100%, PPV of 100%, and NPV of 66.67%. The overall diagnostic accuracy of MDCT for the detection of esophageal varices was 98.97%.

## Discussion

Portosystemic collaterals that develop around the esophagus in cirrhotic patients with portal hypertension are called esophageal varices. Each year, cirrhotic patients develop esophageal varices in 3%–12% of patients, and progression from small to large varices is noted in 8%–12% of patients [10]. Spontaneous regression of small esophageal varices can only be observed if the cause of cirrhosis is removed, such as abstinence from alcohol [11]. A major complication of chronic liver disease is variceal bleeding, which is associated with significant morbidity and mortality. In order to reduce morbidity and mortality from haemorrhage of esophageal varices, it is of significant importance to detect in a timely manner esophageal varices in cirrhotic patients. Mortality of 30%–50% of cirrhotic patients within six weeks of the first variceal bleed was noted in a 15-year data set, and among the survivors, 60% had repeat haemorrhage and mortality of 30% was observed in the following year.

The rates of hospitalization, over all mortality and rebleeding rate secondary to esophageal varices have decreased to 14.2%, 33.5%, and 29%, respectively, due to recent advancements in medical science in the diagnosis and treatment of esophageal varices [12]. Treatment with β-blockers can diminish the probability of bleeding by 50% in patients with medium and large varices by reducing the venous pressure [13]. Alternatively, preventive endoscopic ligation is also used to eradicate the varices. Esophagogastroduodenoscopy (EGD) is expensive and invasive in comparison to CT in detection of esophageal varices, thus limiting its utility as a screening test [14].

In 2007, the American Association for the Study of Liver Diseases (AASLD) stated that screening EGD for the diagnosis of esophageal and gastric varices is recommended when the diagnosis of cirrhosis of liver is made according to the AASLD guidelines. The Baveno IV International Consensus Workshop on portal hypertension also recommended that all cirrhotic patients should be screened for the presence of esophageal varices when liver cirrhosis is diagnosed [15]. During endoscopy, the cases with erythrogenic ﬁndings (especially the red color – ‘RC’ sign) are known to be liable to bleeding [16]. The radiological diagnosis of esophageal varices in cirrhotic patients by CT scan have been described in few studies [17]. A majority of the cirrhotic patients undergo CT evaluation for screening of hepatoma and evaluation of other associated complications. Thus, CT could provide an opportunity to evaluate esophageal varices without additional cost, inconvenience or hazards as compared to invasive endoscopy [17]. For example, a six-monthly CT scan is advised for the screening of hepatocellular carcinoma (HCC) in patients awaiting liver transplantation and in whom variceal screening is especially crucial [18]. CT thus improves the cost-effectiveness in this scenario by allowing evaluation of both HCC and esophageal varices. Moreover, the evaluation of esophageal varices on CT does not require much effort or additional time for the radiologists. CT, therefore, offers a major theoretic advantage over endoscopy in terms of cost-effectiveness especially in countries like Pakistan with scarce health care resources.

In Pakistan, at present, endoscopy is the most commonly used modality for screening of esophageal varices. On the other hand, recent studies in Western literature have attempted to identify high-risk patients using noninvasive measures of portal hypertension such as serum analysis, sonographic spleen size, or both [19]. However, the utility of these models have been debated and the models are not used routinely. The role of computed tomography (CT) or multidetector computed tomography (MDCT) in the detection of gastroesophageal varices before treatment has been reported in a few recent studies. Willmann et al. compared MDCT versus endoscopic ultrasound (EUS) for the detection of varices of the cardiac region. They found that the diagnostic capability of MDCT was equal to that of EUS in distinguishing submucous from perigastric varices [20]. Matsumoto et al. compared the utility of MDCT-portography with conventional angiography in patients with gastric fundus varices [21]. They also reported the comparable utility of MDCT. There have also been some reports about evaluating esophageal and other varices using helical CT and magnetic resonance imaging. Our study results are comparable with the results of these studies, with high sensitivity and specificity for MDCT in the detection of esophageal varices.

There are some limitations in our study. First, the CTs were performed for the screening of hepatoma and, therefore, the CT protocol was not specifically for the evaluation of esophageal varices. Optimization of the CT protocol using thinner slice thickness and a negative oral contrast agent could improve the efficacy of CT in diagnosing esophageal varices. Second, there was a variable time interval between endoscopy and CT (maximum up to four weeks). Therefore, the interval progression or regression of disease cannot entirely be ruled out, although it is highly unlikely given the natural course of esophageal varices. However, caution should be used as variceal size and shape may change secondary to esophageal distention, peristalsis, and hemodynamic changes, such as body fluid status, which vary with time. Further, endoscopy has considerable inter- and intraobserver disagreement, limiting its role as the reference standard and, therefore, the CT correlation only to endoscopy might have limited utility. Correlation of clinical outcomes (e.g. prediction of hemorrhage) with CT variceal grading would have been useful, but this was not possible as the patients were treated appropriately based on concurrent endoscopic findings.

## Conclusions

Our study shows that MDCT is a reliable noninvasive tool for the detection of esophageal varices in clinically suspected cases. We did not evaluate the role of MDCT in grading esophageal varices; therefore, further studies are needed to determine its role as a screening tool for the detection and grading of esophageal varices. Moreover, the use of an optimized CT protocol may further increase the CT accuracy, allowing it to be used as an important alternative or adjunct to endoscopic screening and surveillance.
